# Evaluation of a novel cuffless photoplethysmography-based wristband for measuring blood pressure according to the regulatory standards

**DOI:** 10.1093/ehjdh/ztae006

**Published:** 2024-02-08

**Authors:** Mariska van Vliet, Stefan H J Monnink, Mathijs J Kuiper, Jan C Constandse, Dieke Hoftijzer, Eelko Ronner

**Affiliations:** Department of Cardiology, Reinier de Graaf Hospital, Reinier de Graafweg 5, 2625 AD Delft, The Netherlands; Department of Cardiology, Reinier de Graaf Hospital, Reinier de Graafweg 5, 2625 AD Delft, The Netherlands; Department of Cardiology, Reinier de Graaf Hospital, Reinier de Graafweg 5, 2625 AD Delft, The Netherlands; Department of Cardiology, Reinier de Graaf Hospital, Reinier de Graafweg 5, 2625 AD Delft, The Netherlands; Department of Cardiology, Reinier de Graaf Hospital, Reinier de Graafweg 5, 2625 AD Delft, The Netherlands; Department of Cardiology, Reinier de Graaf Hospital, Reinier de Graafweg 5, 2625 AD Delft, The Netherlands; Corsano Health B.V., Wilhelmina van Pruisenweg 35, 2595 AN The Hague, The Netherlands

**Keywords:** Evaluation, Invasive, Blood pressure, Photoplethysmography, Continuous monitoring, Wearable diagnostics

## Abstract

**Aims:**

Elevated blood pressure (BP) is a key risk factor in cardiovascular diseases. However, obtaining reliable and reproducible BP remains a challenge. This study, therefore, aimed to evaluate a novel cuffless wristband, based on photoplethysmography (PPG), for continuous BP monitoring.

**Methods and results:**

Predictions by a PPG-guided algorithm were compared to arterial BP measurements (in the sub-clavian artery), obtained during cardiac catheterization. Eligible patients were included and screened based on AAMI/European Society of Hypertension (ESH)/ISO Universal Standard requirements. The machine learning-based BP algorithm required three cuff-based initialization measurements in combination with ∼100 features (signal-derived and patient demographic-based). Ninety-seven patients and 420 samples were included. Mean age, weight, and height were 67.1 years (SD 11.1), 83.4 kg (SD 16.1), and 174 cm (SD 10), respectively. Systolic BP was ≤100 mmHg in 48 samples (11%) and ≥160 mmHg in 106 samples (25%). Diastolic BP was ≤70 mmHg in 222 samples (53%) and ≥85 mmHg in 99 samples (24%). The algorithm showed mean errors of ±3.7 mmHg (SD 4.4 mmHg) and ±2.5 mmHg (SD 3.7 mmHg) for systolic and diastolic BP, respectively. Similar results were observed across all genders and skin colours (Fitzpatrick I-VI).

**Conclusion:**

This study provides initial evidence for the accuracy of a PPG-based BP algorithm in combination with a cuffless wristband across a range of BP distributions. This research complies with the AAMI/ESH/ISO Universal Standard, however, further research is required to evaluate the algorithms performance in light of the remaining European Society of Hypertension recommendations.

**Clinical trial registration:**

www.clinicaltrials.gov, NCT05566886.

## Graphical Abstract

**Graphical abstract:**
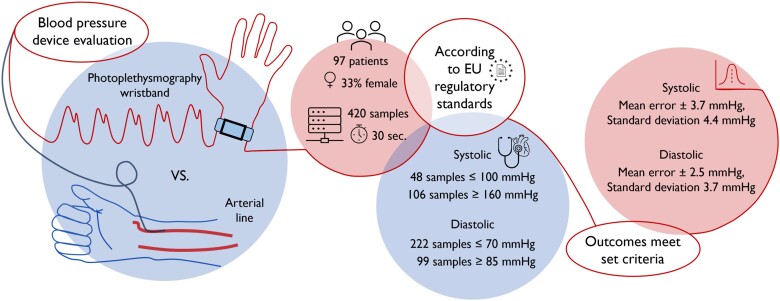

## Introduction

Blood pressure (BP) monitoring is essential in clinical and ambulatory care settings for accurate diagnosis and management of various medical conditions. However, current BP measurement methods encounter challenges, including the fact that it is time consuming, along with record-keeping difficulties. The gold-standard for BP measurement is intra-arterial measurement, however, the use of uncomfortable indwelling catheters poses risks to vulnerable patients.^1^ Hospital cuff BP measurement can be influenced by confounding factors like isolated office hypertension and masked hypertension.^2,3^ Additionally, the principle of vessel compression and subsequent blood flow alterations used in cuff BP measurements does not correlate well with invasive measurements in conditions such as high BP or widespread vessel atherosclerosis.^4^ In ambulatory care, the sporadic nature of measurements may fail to capture true BP patterns over time, hindering optimal management of high BP.

Therefore, there is an unmet need for a non-invasive method of BP measurement that meets the high level of quality required for clinical care, combined with higher measurement frequencies, high accuracy, and ease of use. To this end, the photoplethysmography (PPG) technique has recently emerged as a potential alternative to cuff BP monitors, showing promise in meeting these requirements.^5^ However, despite its potential, no PPG-based BP monitors have met strict care standards and they have not been implemented in clinical practice thus far.

Our study aims to fulfil this need by evaluating the feasibility and accuracy of a cuffless wristband (CardioWatch 287–2, Corsano Health, The Hague, The Netherlands) in combination with a PPG-guided BP-algorithm for continuous BP measurement. Eventually, the aim is to overcome the limitations of cuff BP measurements and to test whether such a PPG-based wristband is able to meet the Association for the Advancement of Medical Instrumentation/European Society of Hypertension/International Organization for Standardization (AAMI/ESH/ISO) Universal Standard requirements.^6,7,8^ Therefore, by evaluating the wristband in light of the AAMI/ESH/ISO Universal Standard requirements for BP monitoring, our research contributes to the advancement of BP measurement practices, ultimately enhancing patient care and outcomes in both clinical and ambulatory settings by improving the quantity and accuracy of data available for treatment optimization.

## Methods

### Study design

The present study is a cross-sectional, single-centre, single-arm study conducted at the Reinier de Graaf Gasthuis (Delft), a teaching hospital in The Netherlands. This study followed an earlier algorithm development trial and was registered under NCT05566886 (ClinicalTrials.gov) and NL80236.000.22 (ToetsingOnline.nl). The study adhered to the principles outlined in the Declaration of Helsinki and received approval from the regional Dutch medical ethical committee.

The AAMI/ESH/ISO Universal Standard used to evaluate the BP monitoring performance of the PPG-based wristband includes ISO 81060-2:2019+A1:2020^6,7^ and the AAMI/ESH/ISO collaboration statement.^8^

### Screening and enrolment

For the populations size the AAMI/ESH/ISO Collaboration Statement recommendations were followed. Therefore, a total of 85 patients for BP accuracy testing with adequate statistical power. A drop-out of 15% was expected due to difficulties with obtaining valid measurement pairs during short and dynamic catheterization exams. Accordingly, the sample size was 100 subjects.

Patients were screened for inclusion and exclusion criteria (*Table 1*). Informed consent was obtained before any study interventions. Patient demographics and medical history were collected during the screening process. Additionally, measurements were taken to determine the patient's arm circumference and mean lateral BP difference, following the recommendations of ISO 81060-2:2019+A1:2020. The diagnostic angiography procedures were performed in a non-interventional cardiology centre, with a triage system to bypass ST elevation patients to interventional centres. As a result, only patients who were hemodynamically stable were included in this research. Patients were excluded if they had a documented diagnosis of persistent arrhythmias or severe tachycardia, or if these conditions were detected during the heart catheterization procedure.

**Table 1:** Trial in- and exclusion criteria

Inclusion criteria	Exclusion criteria
≥18 years old; Arterial line as part of standard care; Able to provide consent.	Unable to wear the CardioWatch 287-2; Unable or not willing to sign informed consent; Significant mental or cognitive impairment; No suitable entry site for the invasive arterial line; Additional conditions provided by ISO 81060-2:2019+A1:2020 Pregnancy; Persistent arrhythmia(‘s) (atrial/ventricular); Severe tachycardia (>120 bpm); Peripheral artery disease. Arm circumference not within cuff range (22–42 cm) Lateral systolic blood pressure difference greater than 15 mmHg Lateral diastolic blood pressure difference greater than 10 mmHg

## Devices

### Investigational product

The CardioWatch 287-2 (CW2) is a rechargeable, wearable device that monitors multiple vital signs, including heart rate, heart rate variability (interbeat intervals), electrocardiogram (ECG), saturation (SpO2), respiration rate, core body temperature, activity, and sleep. It utilizes PPG to perform measurements on the wrist, combining signals from light sources, light sensors, electrodes, and an accelerometer. BP is determined from the PPG signal by means of an artificial intelligence model. This algorithm uses three cuff-based initialization measurements in combination with ∼100 features. Initialization measurements are performed using a validated conventional automated BP cuff (model TMB-2084-A, Zhongshan Transtek Eelctronics Co., Ltd).^9^

To provide a valid prediction, a combination of statistical features, time and frequency domain features, demographic features, first/second derivative features, width-related PPG features, and features from the PPG signal are used in combination with the initialization measurements. Demographic features included the patient’s height, weight, gender, age, and body mass index (BMI). This collection of ∼100 features was chosen after a thorough literature study on features that affect BP.

The intended use of the CW2 BP-algorithm is to provide a single BP reading every 30 min. Nevertheless, due to the use of continuous PPG data BP measurements are possible at various time intervals. As the objective of this study was to evaluate the performance of the CW2 BP-algorithm in light of EU regulatory standards, here, the device was used to output a single BP reading across a 30 s window.

### Non-investigational (reference) product

The Fysicon QMAPP® Hemodynamic Monitoring module (Fysicon B.V., Oss, the Netherlands) was used to record the reference invasive BP measurements in the sub-clavian artery. The QMAPP® device is both EU-Medical Device Regulation (MDR) and US-Food and Drug Administration (FDA) approved, providing accurate raw data with a high sampling frequency.^10^ The validated accuracy and sampling rates of the QMAPP® system are as follows:

- Invasive BP sampled at 100 Hz with an accuracy of ± 1 mmHg.
- Non-invasive BP with an accuracy of ± 5 mmHg.

### Measurements

**Figure 1:**
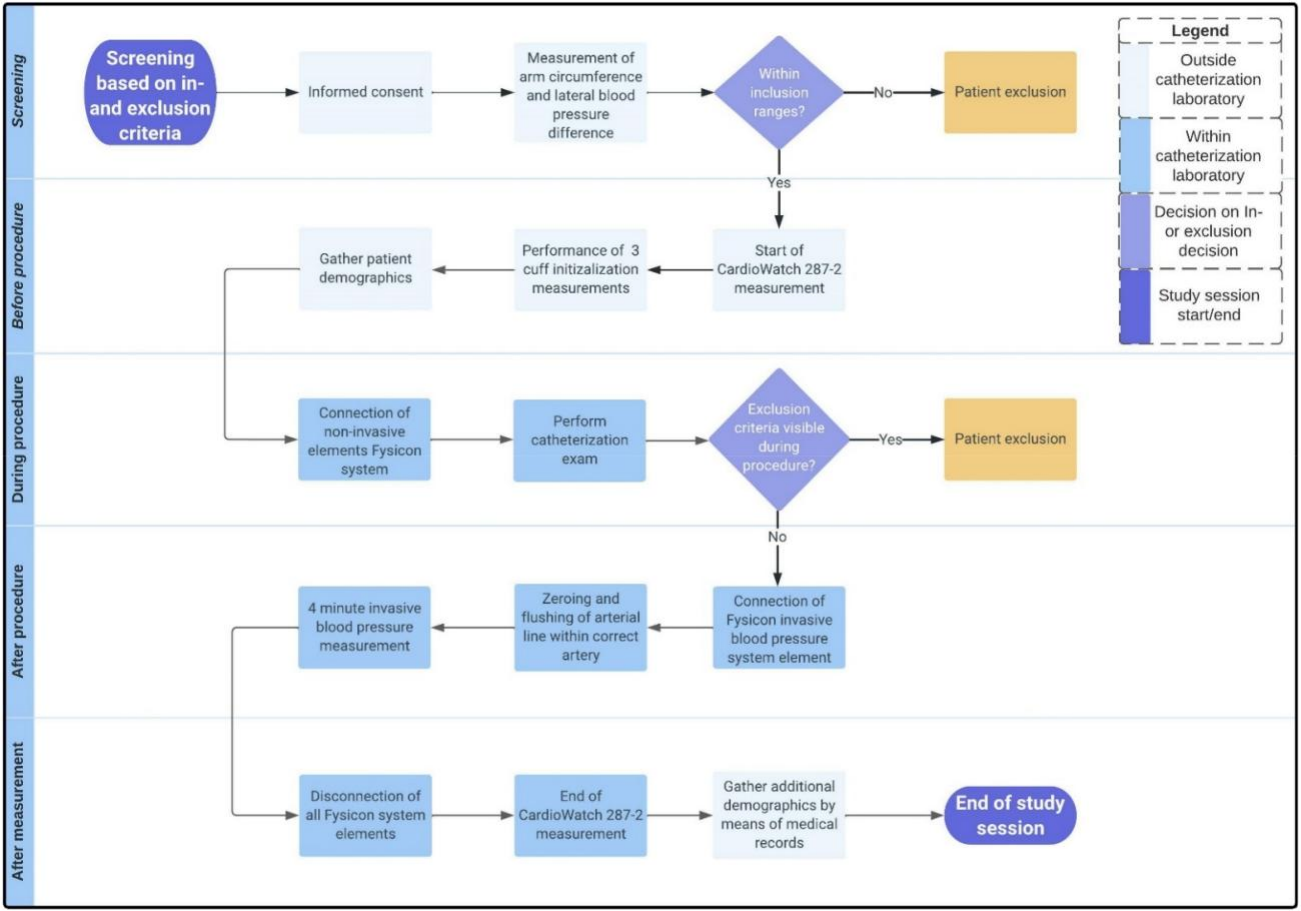
Overview of study procedures.

Study interventions were conducted before, during, and immediately following cardiac examination in the catheterization laboratory. An overview of the study procedure is presented in *Figure 1* and a step-by-step summary of all study interventions is provided in Appendix 1.

In short: First, the CW2 measurement was initiated before the invasive procedure. During this initiation, three cuff BP measurements were performed on the non-CW2 arm to allow for BP-algorithm initialization. After the invasive procedure, a 4-min continuous invasive BP reference measurement within the sub-clavian artery was conducted using the 6 French diameter arterial line. These measurements could be compared to the CW2 BP measurements which were performed simultaneously on the contralateral arm. The entire study procedures were carried out during this single visit, with no follow-up.

All cuff BP measurements adhered to the recommendations outlined by the American Heart Association and ISO 81060-2:2019+A1:2020.^2,6,7^ The cuff BP measurements were performed with patients at rest, without speaking, legs uncrossed, and with their back and cuffed arm supported. Additionally, a time interval of 1–2 min was maintained between consecutive cuff BP measurements.

### Study endpoints

The primary endpoints of this study were to meet the AAMI/ESH/ISO Universal Standard requirements. These endpoints included:

1. Mean error and standard deviation (SD) for systolic and diastolic BP, individually, for a comparison between CW2 BP measurements and invasive reference BP measurements [adjusted for lateral difference (LD)].
2. The number of absolute BP differences between CW2 BP measurements and invasive reference BP measurements within 5, 10, and 15 mmHg, along with the corresponding standardized Bland–Altman scatterplots.

The secondary endpoints of the study involved analysing the results of the primary endpoints based on gender category (male/female) and skin colour categories (Fitzpatrick I-VI).

### Statistical design and analysis

Mean error and SD for systolic and diastolic BP individually were calculated in accordance with EU regulatory standards. The reference systolic BP was defined as the range of ±1 experimental SD around the mean value of the invasive BP values obtained during a 30 s window. The reference diastolic BP was defined in the same way. If the average BP value obtained by the CW2 across the corresponding 30 s window lay within the range of the invasive reference BP an error of 0 mmHg (0 kPa) was assigned. If the average value obtained from the CW2 determination lay outside the range of the invasive reference BP, the adjacent limit of the reference BP was subtracted from the CW2 determination. That difference represented the error for this specific determination.

Using this information, the arithmetic mean of the errors and its experimental SD were determined for the systolic and diastolic BP individually [Appendix 2, Formula (1) and (2)]. Since the opposite limb was used as a reference, the results were corrected for the LD [Appendix 2, Formula (A3), (A4), and (A5)]. In order to comply with the requirements of ISO 81060-2:2019+A1:2020 the resulting arithmetic mean of the error and its experimental SD were required to be no greater than ±5.0 mmHg (±0.67 kPa), and 8.0 mmHg (1.07 kPa), respectively.

Next to the calculation of the mean error and its experimental SD the number of absolute BP differences within 5, 10, and 15 mmHg was provided as well as corresponding standardized Bland–Altman scatterplots, in line with the AAMI/ESH/ISO collaboration statement requirement.

## Results

### Patient characteristics

A total of 124 subjects who underwent heart catheterization as part of their standard care between October 2022 and February 2023 were screened for inclusion in the study. 100 patients were included. The additional 24 patients that were screened for inclusion were replaced due to the following reasons:

- Systolic or diastolic LD higher than required by ISO81060:2-2019 + A1:2020 (*n* = 8)
- Arm circumference not within cuff range (*n* = 1)
- Atrial fibrillation or other severe arrhythmia (*n* = 5)
- Peripheral artery disease (*n* = 3)
- Unforeseen difficulties during heart catheterization leading to time shortage (*n* = 2)
- Other medical reasons (e.g. patient in isolation for infection) (*n* = 1)
- Technical difficulties (measurement not started) (*n* = 1)
- Catheterization procedure cancelled or postponed (*n* = 3)

Retrospectively, data from three individuals were excluded from the analysis due to overdamping of the invasive reference measurement. As a result, data from 97 patients was used for the analysis, resulting in a total of 420 measurement pairs. An overview of the characteristics of these patients is provided in *Table 2*. Lateral differences ranged from 0.00 to 14.70 and 0.00 to 8.30 for systolic BP and diastolic BP, respectively. Mean LD for systolic BP was 4.5 (SD 3.0) mmHg and 2.7 (SD 2.1) mmHg for diastolic BP.

**Table 2:** Patient characteristics

Age (years)	67.1 (SD 11.1)
Gender (female)	32 (33%)
Weight (kilograms)	83.4 (SD 16.1)
Height (centimetres)	174.1 (SD 10.0)
Diabetes (*n*)	21 (21.6%)
Previous myocardial infarction (*n*)	28 (28.9%)
Coronary revascularization in past or recommended (*n*)	29 (29.9%)
Skin colour (Fitzpatrick)^11^	Class I–II: 78 (80.4%)
	Class III–IV: 16 (16.5%)
	Class V–VI: 3 (3.1%)
Arm hair density^12^	Class Nill/Sparse: 56 (57.7%)
	Class Moderate: 35 (36.1%)
	Class High: 6 (6.2%)

### Measurement characteristics

Mean damping coefficient and resonant frequency of the reference invasive BP monitoring equipment were 0.38 (range 0.23–0.57) and 12.11 Hz (range 6.48–19.98 Hz), respectively, complying with the dynamic requirements proposed by Gardner et al.^13^ in all but three cases. These three cases were excluded from the analysis.

Invasive BP distributions were in line with AAMI/ESH/ISO Universal Standard recommendations and are provided in *Table 3*.

**Table 3:** Invasive BP distribution for all patients according to AAMI/ESH/ISO universal standard recommendations

ISO 81060-2:2019+A1:2020^6,7^	AAMI/ESH/ISO Collaboration Statement^8^
Systolic blood pressure	Systolic blood pressure
48 samples (11%) ≤ 100 mmHg	12 patients (12%) ≤ 100 mmHg
106 samples (25%) ≥ 160 mmHg	30 patients (31%) ≥ 160 mmHg
	52 patients (54%) ≥ 140 mmHg
Diastolic blood pressure	Diastolic blood pressure
222 samples (53%) ≤ 70 mmHg	20 patients (21%) ≤ 60 mmHg
99 samples (24%) ≥ 85 mmHg	5 patients (5%) ≥ 100 mmHg
	22 patients (23%) ≥ 85 mmHg

### Primary endpoints

**Figure 2:**
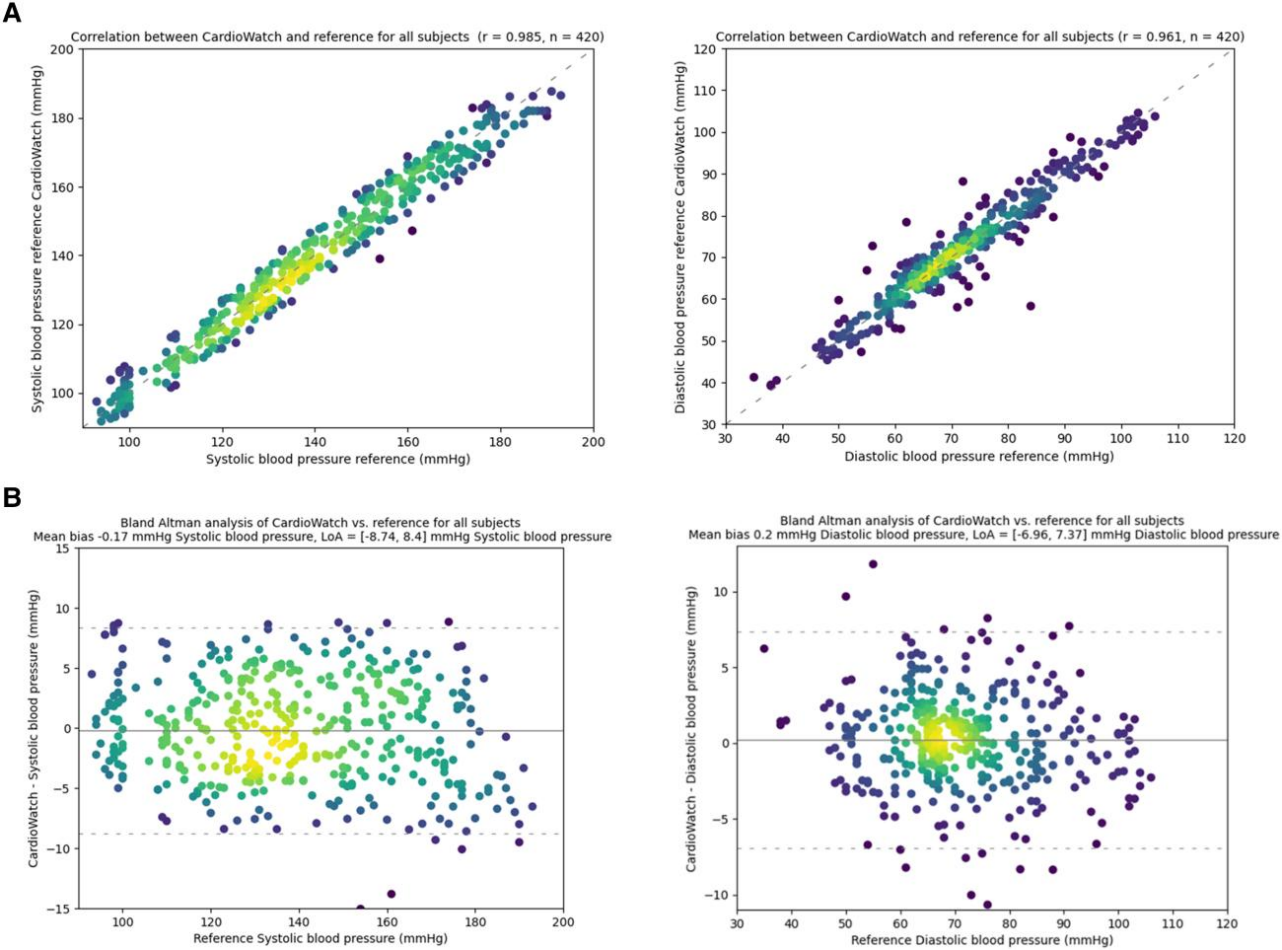
(*A*) Correlation of CW2 BP with reference (fysicon), BP. (*B*) Bland–Altman plot comparing the CW2 Software-derived BP and the invasive Fysicon reference BP pooled over all subjects. Solid lines represent bias and dashed lines represent the limits of agreement.

The mean error and corresponding SD of the differences between CW2 and reference invasive BP measurements was ±3.7 mmHg (SD 4.4 mmHg) and ±2.5 mmHg (SD 3.7 mmHg) for systolic and diastolic BP, respectively. The pooled Bland–Altman plots for the CW2 BP and the invasive Fysicon BP reference are provided in *Figure 2*.

The probability of a mean error ≤10 mmHg between CW2 BP measurements and invasive reference BP measurements was estimated to be 100% for systolic and 99% for diastolic BP. The percentage of absolute BP differences between CW2 and the invasive reference within 5, 10, and 15 mmHg are provided in *Table 4*.

**Table 4:** Percentage of absolute blood pressure differences between CW2 and invasive reference within 5, 10, and 15 mmHg

	≤5 mmHg (%)	≤10 mmHg (%)	≤15 mmHg (%)
Systolic blood pressure (mmHg)	86.4	100	100
Diastolic blood pressure (mmHg)	93.3	99	99.3

### Secondary endpoints

*Table 5* provides the mean error and corresponding SD of the differences between CW2 and reference invasive BP measurements per gender category. More samples were collected for males than females (*n* = 282 vs. *n* = 138). The mean error and SD were largest for male systolic BP, ± 3.8 mmHg (SD 4.6 mmHg).

**Table 5:** Mean and standard deviation of the differences between CW2 and reference invasive BP measurements per gender category

	Male	Male	Female	Female
	Systolic blood pressure (*n* = 282)	Diastolic blood pressure (*n* = 282)	Systolic blood pressure (*n* = 138)	Diastolic blood pressure (*n* = 138)
Mean error	3.8 mmHg	2.5 mmHg	3.5 mmHg	2.6 mmHg
Standard deviation	4.6 mmHg	3.5 mmHg	4.1 mmHg	4.1 mmHg

*Table 6* provides the mean error and corresponding SD of the differences between CW2 and reference invasive BP measurements per skin colour category according to the Fitzpatrick scale. The largest number of samples was collected for Fitzpatrick scale II (*n* = 313), followed by Fitzpatrick III (*n* = 76). Systolic BP of Fitzpatrick II yielded the largest mean error and SD, ± 3.7 mmHg (SD 4.5 mmHg).

**Table 6:** Mean and standard deviation of the differences between CW2 and reference invasive BP measurements per skin colour category according to Fitzpatrick scale

	Fitzpatrick I	Fitzpatrick I
	Systolic blood pressure (*n* = 12)	Diastolic blood pressure (*n* = 12)
Mean error	3.2 mmHg	1.6 mmHg
Standard deviation	3.6 mmHg	2.1 mmHg
	Fitzpatrick II	Fitzpatrick II
	Systolic blood pressure (*n* = 313)	Diastolic blood pressure (*n* = 313)
Mean error	3.7 mmHg	2.6 mmHg
Standard deviation	4.5 mmHg	3.9 mmHg
	Fitzpatrick III	Fitzpatrick III
	Systolic blood pressure (*n* = 76)	Diastolic blood pressure (*n* = 76)
Mean error	3.5 mmHg	2.1 mmHg
Standard deviation	4.1 mmHg	2.7 mmHg
	Fitzpatrick IV	Fitzpatrick IV
	Systolic blood pressure (*n* = 6)	Diastolic blood pressure (*n* = 6)
Mean error	2.4 mmHg	1.7 mmHg
Standard deviation	2.0 mmHg	1.7 mmHg
	Fitzpatrick V and VI	Fitzpatrick V and VI
	Systolic blood pressure (*n* = 13)	Diastolic blood pressure (*n* = 13)
Mean error	3.4 mmHg	2.2 mmHg
Standard deviation	3.3 mmHg	2.6 mmHg

## Discussion

The CW2 BP-algorithm mean errors of ±3.7 mmHg (SD 4.4 mmHg) and ±2.5 mmHg (SD 3.7 mmHg) for systolic and diastolic BP, respectively, across the entire study population. Similar results were observed in all study subgroups, confirming the algorithm’s consistency. These results are in accordance with the AAMI/ESH/ISO Universal Standard.^6,7,8^ Consequently, this study, provides initial evidence supporting the feasibility and accuracy of PPG-based BP measurements, potentially making this technique suitable for clinical use in various patient populations and BP ranges.

Compared to the conventional gold standard of ambulatory BP measurement, which is typically confined to a 24-h period and records only a single vital sign, PPG-based wristband BP measurements offer distinct advantages.^2^ Traditional cuff-based BP monitoring often fails to provide optimal visit intervals for effectively tailored therapy. In contrast, the evaluated method allows for frequent BP measurements with minimal intervals as well as extended monitoring periods.

Furthermore, the direct measurement of BP using PPG-technology offers several advantages over traditional cuff-based methods. PPG is not influenced by cuff inflation, limb compression, or cuff size selection, thereby circumventing potential biases and inaccuracies.^14–17^ Consequently, the application of a PPG-based wristband is expected to expand the possibilities for at-home patient monitoring and reduce the need for hospital or ambulatory measurements. Moreover, timely adjustments to medication based on these simple and frequent BP readings can help prevent both low and high BP-related events, ensuring optimal patient care.

To date, only a few studies have investigated the performance of PPG-based BP measurements in the light of AAMI/ESH/ISO Universal Standard. Islam et al.^18^ conducted a meta-analysis on PPG-based BP measuring techniques evaluated between 2012 and 2019. Their findings showed a mean difference of 3.16 ± 4.13 mmHg (range: −1.80 to 13.19 mmHg) for systolic BP and 1.22 ± 2.25 mmHg (range: −1.00 to 5.86 mmHg) for diastolic BP. It is worth noting that all evaluations included in this meta-analysis used cuff-based BP measurements as a reference, while the present study employed arterial reference measurements, as this may provide a more direct representation of the patients BP.^19^ Although cuff and arterial BP measurements are known to deviate for specific BP ranges the results found here align with those presented in the meta-analysis by Islam et al. (2022).^19,20^ This result may be explained by the fact that only the average differences across the entire population were compared whilst approximately half of the patients studied had BPs within ranges that are considered comparable between cuff and arterial measurements (<120/80 mmHg and ≥160/120 mmHg).^20^ A closer comparison to the technique proposed here may be formed by Pellaton et al.^21^ which also used arterial reference measurements. However, their results showed a larger deviation from those presented here, as they achieved mean differences of 0.0 ± 7.1 mmHg for the systolic BP and 0.0 ± 2.9 mmHg for the diastolic BP. This may be due to the fact that this study had a relatively small sample size of 23 subjects compared to the 97 patients included in this research.

Variations in outcomes may also be related to differences in features or underlying algorithms. In this research a collection of ∼100 features was used, including statistical features, time and frequency domain features, demographic features, first/second derivative features, width-related PPG features, and features from the PPG signal. To identify this optimal collection of features the performance of various feature sets was evaluated during internal research. This research also revealed that the use of demographic features alone results in a linear regressor model per patient or patient group, leading to large prediction errors in cases with high inter- and intra-patient variability. This finding is in line with earlier research.^22^

Most of the investigated PPG-based BP measurement devices only allow the measurement of BP.^17^ However, the ability to measure multiple other parameters, such as pulse rate, atrial fibrillation, SpO_2_, breathing frequency, and accelerometer data, alongside BP, could assist in the early detection of various disease states and could facilitate improved guidance of therapies. For instance, medically certified wristband data can be linked to exercise levels, shortness of breath, palpitations, pain, and fatigue, enabling objective, cost-effective, and timely differential diagnostics. Therefore, a device such as the one evaluated in this study may facilitate early intervention and personalized treatment strategies, ultimately improving patient outcomes and reducing healthcare burdens.^23^

Despite the significant findings of this study, it is essential to acknowledge its limitations. Firstly, the study was conducted in a single-centre with a limited number of patients, which may limit the generalizability of the results. For the study size, AAMI/ESH/ISO collaboration statement recommendations were followed, however, this statement is not considerd an invasive reference method. Nonetheless, the invasive ISO 81060-2 protocol only provides a maximum amount of measurements and not a maximum amount of participants. Due to the high amount of cardiac catheterizations and limited additional patient burden a study population of 100 patients was considered ethical. Moreover, this large study population allowed a more thorough evaluation of the performance of the CW2 BP-algorithm across various patient characteristics, as also stated by the AAMI/ESH/ISO collaboration statement.

The diagnostic angiography setting, as well as catheter placement choice, should also be noted, as selection bias may have occurred here. Due to size, the catheter was placed in the sub-clavian artery and not in the brachial artery, where spasm and flow impairment would be more likely. Future studies involving larger, multi-centre cohorts are necessary to validate the findings across diverse populations and healthcare settings. Secondly, the exclusion of patients with arrhythmias, severe tachycardia (>120 bpm), and peripheral artery disease may restrict the applicability of the PPG-based wristband in these specific patient groups. Further research is warranted to assess the performance of these populations. Moreover, the current trial recordings were conducted on patients at rest for a short time period, which may not fully reflect the performance of the wearable device and algorithm in an unsupervised ambulatory setting. Future investigations should evaluate the BP-algorithm under dynamic circumstances over an extended period to establish its reliability and effectiveness in real-world scenarios.

Lastly, it is important to note that the AAMI/ESH/ISO Universal Standard used for evaluating the proposed PPG-based BP measurement technique was not specifically developed for cuffless devices. As such, these standards lack criteria for evaluating the device’s ability to track BP changes within an individual or its measurement stability after initialization.^24,25^

Existing guidelines for the evaluation of (intermittent) cuffless BP devices have been rendered impractical and difficult to implement due to a lack of specificity, specifically concerning the means to induce BP changes.^26^ A new regulatory standard, ISO 81060-3:2022, tailored to the clinical investigation of continuous automated non-invasive sphygmomanometers was published in December 2022.^27^ However, as the CW2 is intended to provide BP determinations at an interval of 30 min during remote patient monitoring this new standard was rendered unapplicable here. Furthermore, this new standard has its own limitations, including concerns about the feasibility of performing long-term intra-arterial reference measurements.^24,28^

In June 2023, after the completion of this study, a new recommendation for the evaluation of intermittent cuffless BP devices was introduced by the ESH.^26^ Although additional evaluations during exercise, sleep, up titration of BP lowering medication, changes in body position, and after long-term monitoring (recalibration period) are recommended, the ESH still refers to the AAMI/ESH/ISO Universal Standard. Therefore, this study, in part, complies with the static state test of the new ESH recommendations. However, as this static test is not specifically recommended for cuff-calibrated devices, the remaining ESH recommendations are currently being tackled in additional studies, such as the RECAMO study (NCT05899959, ClinicalTrials.gov).

## Conclusion

This study provides initial evidence for the accuracy of the combination of a PPG-based wristband and its BP-algorithm against current EU regulatory standards, demonstrating the feasibility of this technique for BP monitoring. The continuous monitoring capabilities, coupled with the potential for real-time analysis and early disease detection, hold great promise for improving patient care and outcomes. However, further research is warranted to address the limitations of this study and evaluate the performance of the PPG-based wristband in light of the remaining ESH recommendations as well as in an ambulatory setting.

## Data Availability

Data will be made available on reasonable request.

## Appendix 1 Study procedures step-by-step

### Before standard invasive procedure

1. Obtaining informed consent.
2. Starting of CW2 measurement.
3. Screening for two additional criteria:
  - Measurement of arm circumference according to ISO 81060-2:2019+A1:2020 instructions (acceptable range: 22–46 cm; exclusion if outside this range).
  - Evaluation of LD in BP through three cuff BP measurements on both arms simultaneously, using two identical automated sphygmomanometers. Exclusion based on ISO 81060-2:2019+A1:2020 limits:
    - Mean LD of the reference systolic BP readings is more than 15 mmHg; or
    - Mean LD of the reference diastolic BP readings is more than 10 mmHg.
4. Conducting three initialization measurements for the CW2 BP-algorithm using a non-invasive cuff measurement on the non-CW2 arm.

### During standard invasive procedure

1. Measure non-invasive BP using the QMAPP BP cuff every 5 min.

### Directly following standard invasive procedure

1. Invasive BP reference measurement:
  - Appropriate measures were taken to remove air bubbles and clots from the system before obtaining the reference measurements.
  - Zeroing of the arterial line reference measurement was performed.
  - A fast flush test was conducted to characterize the resonant frequency and damping coefficient of the reference invasive BP monitoring equipment, meeting the dynamic requirements proposed by Gardner^13^ for each measurement.
  - A 4-min continuous invasive BP reference measurement within the sub-clavian artery was conducted using the arterial line.
    - No non-invasive BP measurements were taken during the invasive measurement to avoid interrupting the CW2 measurement.
2. The CW2 measurement was stopped.

## Appendix 2 Statistical design and analysis formulas

(A1)
$${\bar{x}}_{n}=\frac{1}{n}\times \overset{n}{\underset{i=1}{\sum }}\;x_{i}$$

Formula for calculation of the arithmetic mean of the error (*x_n_*). Where *x_i_* is the error of the *i*th individual determination, *n* is the total number of determination and *i* is the index for the individual determination.

(A2)
$$SD=\sqrt{\frac{1}{n-1}\times \overset{n}{\underset{i=1}{\sum }}\;{\left(x_{i}-{\bar{x}}_{n}\right)}^{2}}$$

Formula for calculation of the experiment SD. Where *x_i_* is the error of the *i*th individual determination, *n* is the total number of determinations, *x_n_* is the arithmetic mean of the error and *i* is the index for the individual determination.

(A3)
$$LD=\frac{1}{3}\times \left(\overset{3}{\underset{i=1}{\sum }}\;p_{i}-\overset{3}{\underset{\;j=1}{\sum }}\;p_{j}\right)$$

Formula for calculation of the LD. Where *i* is the index for the determination with the automated sphygmomanometer on the same limb as the CW2 determinations and j is the index for the reading on the limb used for the reference reading.

(A4)
$$\;x=p_{CW_{L}}-\;p_{REF_{R}}+LD$$

(A5)
$$\;x=p_{REF_{L}}-\;p_{CW_{R}}+LD$$

Formula’s for correcting the error in the case that the CW2 is placed on the left (A4) and right (A5) arm. Where $p_{CW_{L}}$ and $p_{CW_{R}}$ are CW2 blood pressures in the left (L) arm and right (R) arm, respectively.

## References

[1] Scheer B , Perel A, Pfeiffer UJ. Clinical review: complications and risk factors of peripheral arterial catheters used for haemodynamic monitoring in anaesthesia and intensive care medicine. Critical Care 2002;6:199. 12133178 10.1186/cc1489 PMC137445

[2] Muntner P , Shimbo D, Carey RM, Charleston JB, Gaillard T, Misra S, et al Measurement of blood pressure in humans: a scientific statement from the American heart association. Hypertension 2019;73:35–66. 10.1161/HYP.0000000000000087 PMC11409525 30827125

[3] Papadogiannis DE , Protogerou AD. Blood pressure variability: a confounder and a cardiovascular risk factor. Hypertens Res 2010;34:162–163. 21107334 10.1038/hr.2010.223

[4] Bui TV , Picone DS, Schultz MG, Armstrong MK, Peng X, Black JA, et al Comparison between cuff-based and invasive systolic blood pressure amplification. J. Hypertens. 2022;40:2037–2044. 36052526 10.1097/HJH.0000000000003228 PMC7614121

[5] Nitzan M , Slotki I, Shavit L. More accurate systolic blood pressure measurement is required for improved hypertension management: a perspective. Med Devices Evid Res 2017;10:157–163. 10.2147/MDER.S141599 PMC5533571 28769596

[6] ISO 81060-2:2018 [Internet]. Non-invasive sphygmomanometers—Part 2: Clinical investigation of intermittent automated measurement type. 2018 [cited 2023 Jul 26]. Available from: https://www.iso.org/standard/73339.html

[7] ISO 81060-2:2018/AMD 1:2020. Non-invasive sphygmomanometers—Part 2: Clinical investigation of intermittent automated measurement type—Amendment 1. 2020 [cited 2023 Jul 26]. Available from: https://www.iso.org/standard/75432.html

[8] Stergiou GS , Alpert B, Mieke S, Asmar R, Atkins N, Eckert S, et al A universal standard for the validation of blood pressure measuring devices. Hypertension 2018;71:368–374. 29386350 10.1161/HYPERTENSIONAHA.117.10237

[9] Transtek bluetooth blood pressure monitor (ARM) telerpm BPM(BLE) 2022, BP bluetooth [Internet]. 2022 [cited 2023 Nov 3]. Available from: https://www.transtekcorp.com/products/bluetooth-5.0-blood-pressure-monitor-arm-tmb-2084-a/

[10] Fysicon B.V. QMAPP® User manual—QMAPP 2.5—English. Oss, The Netherlands: Fysicon B.V.; 2021, p174–177.

[11] Fitzpatrick TB . The validity and practicality of sun-reactive skin types I through VI. Arch Dermatol 1988;124:869–871. 3377516 10.1001/archderm.124.6.869

[12] von Schuckmann LA , Hughes MC, Green AC, van der Pols JC. Forearm hair density and risk of keratinocyte cancers in Australian adults. Arch Dermatol Res 2016;308:617–624. 27590883 10.1007/s00403-016-1680-5

[13] Gardner RM . Direct blood pressure measurement—dynamic response requirements. Anesthesiology 1981;54:227–236. 7469106 10.1097/00000542-198103000-00010

[14] Bradley CK , Shimbo D, Colburn DA, Pugliese DN, Padwal R, Sia SK, et al Cuffless blood pressure devices. Am J Hypertens 2022;35:380–387. 35136906 10.1093/ajh/hpac017 PMC9088838

[15] Sprafka JM , Strickland D, Gómez-Marín O, Prineas RJ. The effect of cuff size on blood pressure measurement in adults. Epidemiology 1991;2:214–216. 2054405 10.1097/00001648-199105000-00010

[16] Charmoy A , Würzner G, Ruffieux C, Hasler C, Cachat F, Waeber B, et al Reactive rise in blood pressure upon cuff inflation: cuff inflation at the arm causes a greater rise in pressure than at the wrist in hypertensive patients. Blood Press Monit 2007;12:275–280. 17890965 10.1097/MBP.0b013e3282c9ac9a

[17] Veerman DP , van Montfrans GA, Wieling W. Effects of cuff inflation on self-recorded blood pressure. Lancet 1990;335:451–453. 1968178 10.1016/0140-6736(90)90676-v

[18] Islam SM , Chow CK, Daryabeygikhotbehsara R, Subedi N, Rawstorn J, Tegegne T, et al Wearable cuffless blood pressure monitoring devices: a systematic review and meta-analysis. Eur Heart J Digit Health 2022;3:323–337. 36713001 10.1093/ehjdh/ztac021 PMC9708022

[19] Cambiaso-Daniel J , Rontoyanni VG, Foncerrada G, Nguyen A, Capek KD, Wurzer P, et al Correlation between invasive and noninvasive blood pressure measurements in severely burned children. Burns 2018;44:1787–1791. 30153960 10.1016/j.burns.2018.03.001 PMC6387786

[20] Picone DS , Schultz MG, Otahal P, Aakhus S, Al-Jumaily AM, Black JA, et al Accuracy of cuff-measured blood pressure. J Am Coll Cardiol 2017;70:572–586. 28750701 10.1016/j.jacc.2017.05.064

[21] Pellaton C , Vybornova A, Fallet S, Marques L, Grossenbacher O, De Marco B, et al Accuracy testing of a new optical device for noninvasive estimation of systolic and diastolic blood pressure compared to intra-arterial measurements. Blood Press Monit 2020;25:105–109. 31688003 10.1097/MBP.0000000000000421

[22] Finnegan E , Davidson S, Harford M, Watkinson P, Tarassenko L, Villarroel M. Features from the photoplethysmogram and the electrocardiogram for estimating changes in blood pressure. Sci Rep 2023;13:986. 36653426 10.1038/s41598-022-27170-2 PMC9849280

[23] Avolio A , Shirbani F, Tan I, Butlin M. Cuffless blood pressure monitoring and the advent of a new era in medicine and society. In: The Handbook of Cuffless Blood Pressure Monitoring. Sydney, Australia: Macquarie University; 2019. p1–7.

[24] Stergiou GS , Mukkamala R, Avolio A, Kyriakoulis KG, Mieke S, Murray A, et al Cuffless blood pressure measuring devices: review and statement by the European Society of Hypertension Working Group on blood pressure monitoring and cardiovascular variability. J Hypertens. 2022;40:1449–1460. 35708294 10.1097/HJH.0000000000003224

[25] Mukkamala R , Yavarimanesh M, Natarajan K, Hahn J-O, Kyriakoulis KG, Avolio AP, et al Evaluation of the accuracy of cuffless blood pressure measurement devices: challenges and proposals. Hypertension 2021;78:1161–1167. 34510915 10.1161/HYPERTENSIONAHA.121.17747 PMC8516718

[26] Stergiou GS , Avolio AP, Palatini P, Kyriakoulis KG, Schutte AE, Mieke S, et al European Society of Hypertension Recommendations for the validation of cuffless blood pressure measuring devices: European Society of Hypertension Working Group on blood pressure monitoring and cardiovascular variability. J Hypertens. 2023;41:2074–2087. 37303198 10.1097/HJH.0000000000003483

[27] ISO 81060-3:2022. Non-invasive sphygmomanometers—Part 3: Clinical investigation of continuous automated measurement type. 2022 [cited 2023 Jul 26]. Available from: https://www.iso.org/standard/71161.html

[28] Tamura T , Shimizu S, Nishimura N, Takeuchi M. Long-term stability of over-the-counter cuffless blood pressure monitors: a proposal. Health Technol (Berl) 2023;13:53–63. 36713070 10.1007/s12553-023-00726-6 PMC9870659

